# Insect Flight Energetics and the Evolution of Size, Form, and Function

**DOI:** 10.1093/icb/icae028

**Published:** 2024-04-30

**Authors:** Charles-A Darveau

**Affiliations:** Department of Biology, University of Ottawa, 30 Marie Curie, Ottawa, Ontario K1N 6N5, Canada

## Abstract

Flying insects vary greatly in body size and wing proportions, significantly impacting their flight energetics. Generally, the larger the insect, the slower its flight wingbeat frequency. However, variation in frequency is also explained by differences in wing proportions, where larger-winged insects tend to have lower frequencies. These associations affect the energy required for flight. The correlated evolution of flight form and function can be further defined using a lineage of closely related bee species varying in body mass. The decline in flight wingbeat frequency with increasing size is paralleled by the flight mass-specific metabolic rate. The specific scaling exponents observed can be predicted from the wing area allometry, where a greater increase (hyperallometry) leads to a more pronounced effect on flight energetics, and hypoallometry can lead to no change in frequency and metabolic rate across species. The metabolic properties of the flight muscles also vary with body mass and wing proportions, as observed from the activity of glycolytic enzymes and the phospholipid compositions of muscle tissue, connecting morphological differences with muscle metabolic properties. The evolutionary scaling observed across species is recapitulated within species. The static allometry observed within the bumblebee *Bombus impatiens*, where the wing area is proportional and isometric, affects wingbeat frequency and metabolic rate, which is predicted to decrease with an increase in size. Intraspecific variation in flight muscle tissue properties is also related to flight metabolic rate. The role of developmental processes and phenotypic plasticity in explaining intraspecific differences is central to our understanding of flight energetics. These studies provide a framework where static allometry observed within species gives rise to evolutionary allometry, connecting the evolution of size, form, and function associated with insect flight.

Animal size, locomotion, and energy metabolism are closely linked, and these connections are a central theme in integrative comparative biology. The way animals move is a significant factor that drives the evolution of their energetic properties. The size and shape of an animal’s body, along with its movements, affect its musculoskeletal properties and locomotor performance (Biewener 1991; Rome 1992; Alexander 2005; Biewener and Patek 2018). These factors also impact the systems and tissues responsible for energy supply and demand (Weibel 2000; Weibel et al. 2004). The variation in animal body mass explains the bulk of the variation in metabolic rate (MR) during activity (Savage et al. 2004; Weibel et al. 2004; Alexander 2005; Painter 2005; da Silva et al. 2006), which is a well-defined interspecific physiological scaling pattern also termed evolutionary allometry. The mechanistic explanations for MR scaling during locomotion are central to understanding the evolution of form and function. Flying insects can exemplify these connections. I will review the scaling relationship between body size and wing proportion and show how it impacts flight wingbeat frequency (WBF) and MR among insects. Combining a series of studies conducted on bees will also show how we can reconcile evolutionary allometry observed among species with static allometry found within species. This synthesis provides an integrated view of the evolution of form and function associated with insect flight energetics and metabolism.

## Broad-scale comparisons

**Fig. 1.**
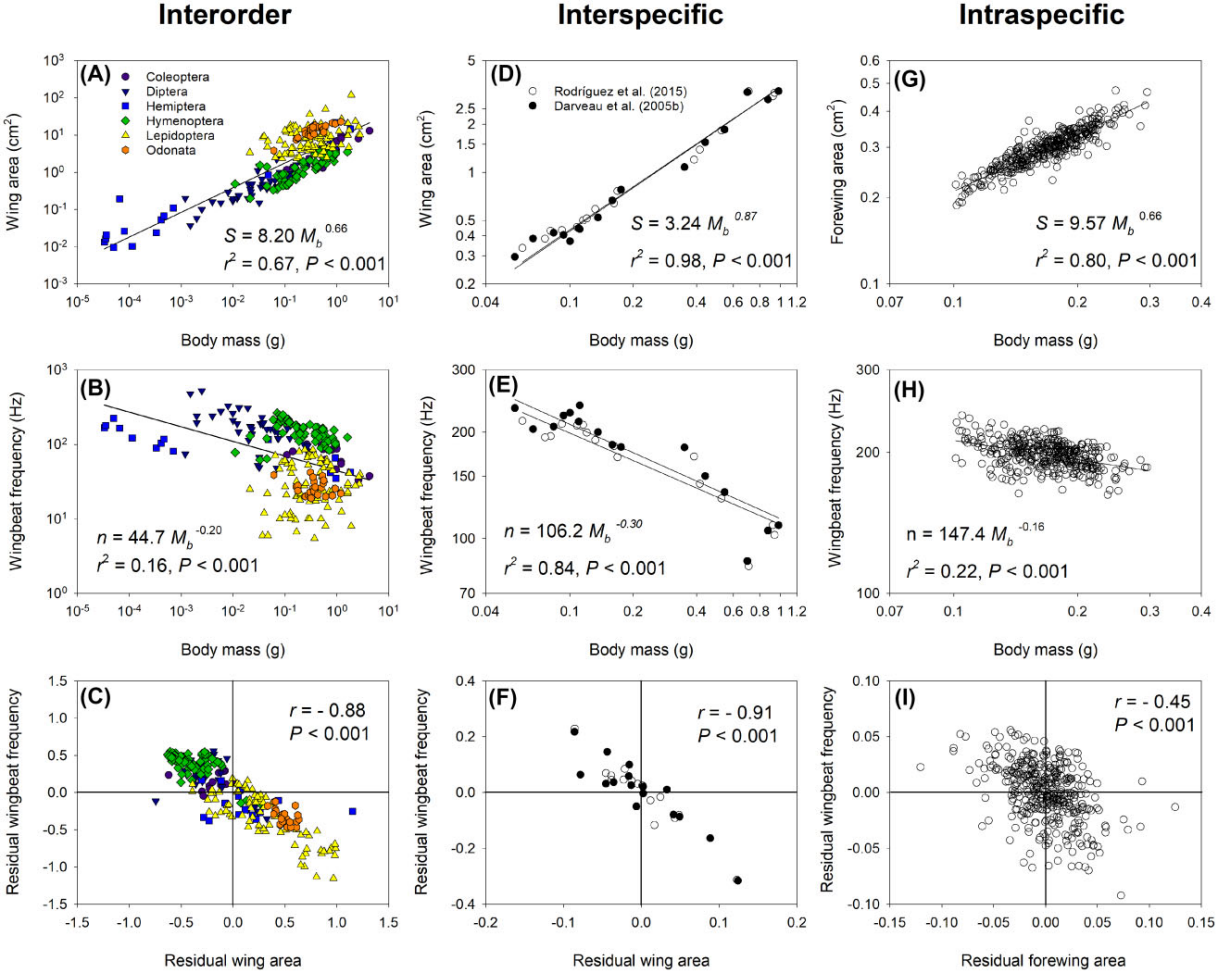
Relationship between body mass, wing area, and wingbeat frequency among insect species from many orders (A−C), closely related species of bees (D−F), and individuals of a bee species (G−I). Interorder variation of insect species compiled by Byrne et al. (1988) shows the body mass dependence of wing area (A) and wingbeat frequency (B). The residual variation obtained from the regressions with body mass is negatively correlated (C), showing that order and species with larger wings for a given size have lower wingbeat frequencies. Interspecific variation among orchid bees is shown for the same species obtained from two data sets (Darveau et al. 2005b; Rodríguez et al. 2015) that show the consistent relationships of wing area (D) and wingbeat frequency (E) with body mass (equation shown for the Darveau et al. 2005b data only). The residual variation from the body mass relationships (F) indicates that species with slightly larger wings for their size have slightly lower wingbeat frequency. At the intraspecific level, workers of the bumble bee species *Bombus impatiens* also show a significant effect of body mass on wing area (G) and wingbeat frequency (H) and a significant negative correlation between residuals obtained from the body mass relationships (I).

Flying insects come in a wide range of sizes, from tiny fairy flies *Tinkerbella nana* or the even smaller *Kikiki huna* at 150 µm in length (Huber and Noyes 2013) to large Atlas moth *Attacus atlas* with wingspans exceeding 25 cm (Deora et al. 2017). Extinct insects exceeded this range, such as the giant dragonfly with wings spanning 70 cm, posing flight energetics challenges (Cannell 2018). This size variation significantly impacts the biomechanics of flight, a subject that continues to challenge biologists (Cheng and Sun 2021). Insects also vary greatly in wing size for a given body mass, such as butterflies and bees of similar body mass, which can vary in wing size by about 70-fold (Corben 1983). The relationship between species’ body mass and wing size can be described with power functions typical of biological allometry (Gayon 2000), using the equation *Y* = *a*X*^b^*, where the wing area of proportionally sized insects would scale with body mass to the 2/3 power. Broad comparisons among insect orders follow this general prediction (Fig. 1A) from data compiled on 160 species (Byrne et al. 1988) and further supported by an independent data set (Tercel et al. 2018). This relationship can be used to predict the general relationship between body mass and flight WBF. Morphological scaling can impact flight WBF variation, a central factor affecting force production and generating lift (Deora et al. 2017). Studies have shown that larger wings for a given size will beat at a lower frequency (Dorsett 1962; Bartholomew and Heinrich 1973; Bartholomew and Casey 1978), also applicable to birds and bats (Corben 1983; Pennycuick 2008; Norberg and Norberg 2012). Such association can be predicted using dimensional analysis, where geometrically similar species are expected to decrease in frequency with increasing size with *b* = −1/6 (Pennycuick 2008; Deakin 2010), which is close to the *b* = −0.21 ± 0.03 (SE) obtained across insect orders (Fig. 1B). WBF variation can be better described as a function of both species’ body mass and wing area (Bartholomew and Casey 1978; Corben 1983; Casey et al. 1985; Darveau et al. 2005b; Ha et al. 2013; Aiello et al. 2021). The variation in frequency not accounted for by body mass, as represented by residual variation from the regression in Fig. 1B, can be explained by the residual variation in wing area among species (Fig. 1C). Thus, WBF variation can be generally described as a function of species body mass and wing area and summarized by the relationship WBF = *K M _b_*^1/2^  *S*^−1^ (Corben 1983; Deakin 2010; Ha et al. 2013), where *K* is a scaling factor, *M _b_* body mass, and *S* wing area, leading to WBF = *K M _b_*  ^−1/6^ for geometrically similar animals where *S* = *M _b_*^2/3^.

The frequency at which an insect beats its wings impacts its MR during flight. The relationship between muscle contraction frequency, power output, and metabolic power input leads to this expectation (Pennycuick and Rezende 1984; Alexander 2005; Medler and Hulme 2009). Early accounts indicate that flying insects do not obey the classic decrease in mass-specific MR during flight (Kammer and Heinrich 1978). However, obtaining enough data to compare the scaling patterns across different insect orders is challenging. Furthermore, insects are a hyperdiverse group, and their various means of staying aloft, capacity for flight, activation patterns of the flight muscles, endothermic capacity, varying abundance of muscles, and body proportions make it challenging to characterize simple scaling patterns (Kammer and Heinrich 1978; Full 1997; Waters and Harrison 2012). Nevertheless, data compiled on over 50 species show that insects follow a hypoallometric relationship where whole-animal MR scales with a 0.86 power, but species weighing less than 10 mg have lower flight MR (Niven and Scharlemann 2005). Hovering insects also follow hypoallometric scaling only after reaching a threshold mass of 58 mg (Duell et al. 2022). With flight muscle as the primary contributing tissue to flight MR, the hypoallometric scaling reported by Niven and Scharlemann can be expressed on a mass-specific basis to obtain an exponent value of *b* = −0.14, which is not too far from the decrease in frequency. Still, such broad comparisons include many factors contributing to the variability observed. The wings’ beating frequency and mass-specific MR generally scale similarly due to the relationship between muscle contraction frequency and muscle-specific metabolic power. However, this comparison involves many other physiological properties that may complicate the straightforward relationship. Therefore, comparing species within a narrower taxonomic group can remove some of this complexity and better resolve the connections between flight form and function.

## Phylogenetically informed analysis within an insect family

The general framework used to predict the impact of body and wing size on insect flight energetics can be used to investigate the correlated evolution of flight form and function. Orchid bees have provided many insights into insect flight energetics (Casey et al. 1985; Casey and Ellington 1992; Askew et al. 2010), and we further expanded and incorporated phylogenetically informed analysis in their study. This group of hymenopterans spans a 20-fold range in body mass and shows a WBF scaling exponent of −0.30 (Fig. 1E), steeper than the −1/6 value predicted for geometrically similar insects. Disproportional changes in the wing area of this group of bees can explain the steeper relationship with body mass. The wing area increases hyperallometrically with an exponent of 0.87 (± 0.04) instead of the 2/3 expected for geometrically similar species (Fig. 1D). Substituting the wing area exponent value in Deakin’s equation, or wing surface and length in Pennycuick’s, predicts a steeper scaling exponent for WBF of −0.37 and −0.30, respectively, which is in line with the observed −0.30 (± 0.04). The departure from the scaling pattern with a steeper relationship is adequately predicted, given the disproportionate increase in wing size in this lineage of bees.

**Fig. 2.**
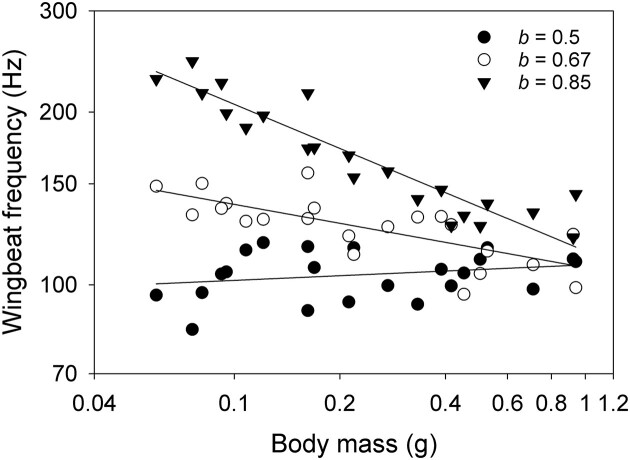
Simulated effect of varying wing area allometry and its impact on the wingbeat frequency relationship with body mass. Simulations were conducted using the body mass range from orchid bees shown in Fig. 1 and the empirical relationship, WBF = 295.5 *M _b_*^0.45^  *S*^−0.87^. The wing area term *S* was substituted for the relationship *S* = 3.24 *M _b_*^0.50^, *S* = 3.24 *M _b_*^0.67^, or *S* = 3.24 *M _b_*^0.85^. Simulations conducted with GraphPad Prism added a 10% relative Gaussian random error to the predicted values. Non-linear regression analysis fitting a power function was conducted, and the results reported are the mean estimates of 1000 Monte Carlo simulations. ●*b* = 0.5: 109 *M _b_*^0.03^, *r*^2^ = 0.09; ○*b* = 0.67: WBF = 98 *M _b_*^−0.17^, *r*^2^ = 0.66; ▼ *b* = 0.85: WBF = 103 *M _b_*^−0.31^, *r*^2^ = 0.87.

Despite the much smaller residual variation around the regression lines, size-independent deviations in wing proportions explain most of the remaining variation. Species with larger wings for a given size beat them at a lower frequency. The coefficient of determination (*r*^2^) explaining WBF is 0.84 when body mass alone is used (WBF = 106.3 *M _b_*^−0.30^) and further increases to 0.97 when wing area is added to the model (WBF = 295.5 *M _b_*^0.45^  *S*^−0.87^). Scaling equations are useful predictive tools, but deviations from the general patterns also help identify the functional basis of such deviations. A lack of scaling effect can also tell us about the functional associations at play. In another lineage of bees, the stingless bees, different scaling patterns are observed where the forewing area scales hypoallometrically with an exponent of 0.5 (0.57 for total wing area) (Duell et al. 2022). The prediction is that WBF should scale with body mass with a shallow slope or not at all, which is what they observed. Using the equation obtained on orchid bees and simply substituting the wing area *S* with body mass scaling exponent between *b* = 0.5, 0.67, and 0.85 in simulated data sets, the scaling of WBF can be removed entirely (Fig. 2). Despite the contrasting results with distinct scaling patterns, the same associations between form and flight function can be used to explain differential scaling.

**Fig. 3.**
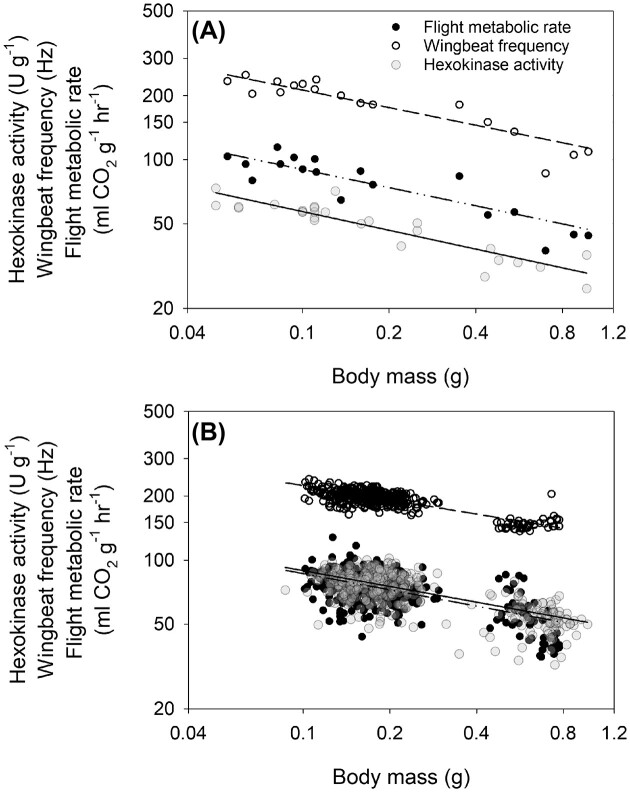
Parallel scaling of hovering flight wingbeat frequency, mass-specific metabolic rate, and the activity of the glycolytic enzyme hexokinase in the flight muscle among orchid bee species (A) and bumblebee (*Bombus impatiens*) individuals (B). The interspecific allometry of all species sampled in Darveau et al. (2005b) shows the same scaling exponent *b* = −0.31 flight wingbeat frequency and metabolic rate, and *b* = –0.33 for the activity of hexokinase (Darveau et al. 2005a). For the intraspecific allometry obtained from Billardon and Darveau (2019), the regressions presented are for workers only forming the cluster of smaller-sized individuals with a scaling exponent of *b* = −0.16 for both wingbeat frequency and metabolic rate, and *b* = −0.14 for hexokinase activity. The cluster of larger individuals are queens that fall on the predicted line from the workers’ relationship.

Several studies on bees and moths have highlighted the relationship between species’ flight MR, size and wing proportions, and flight kinematics. Differences in flight MR in moth species ranging 10-fold in body mass have been used to estimate power output and components (Casey 1981). Similar associations were uncovered in the orchid bees (Casey et al. 1985). Using a phylogenetically informed analysis with more species, we see that flight MR scaling parallels WBF scaling with an exponent of −0.31 (Fig. 3A). In this group of closely related species, flight MR is a direct function of flight WBF, which is, in turn, a function of the species’ body and wing size. All else being equal, deviation in wing proportion scaling should ultimately similarly impact flight MR scaling as simulated for WBF (Fig. 2). The case of stingless bees with hypoallometric scaling of wing size shows such deviation where flight MR is independent of body mass. However, the additional differences in endothermic capacity among species within this lineage may also contribute to the variation in flight MR (Duell et al. 2022). In orchid bees, slight deviations in species’ thoracic surface temperature explain part of the variation in MR (Rodríguez et al. 2015). Additionally, variations in body proportions, such as flight muscle mass, are known to affect flight energetics and should be accounted for in such comparisons (Marden 2000). Further studies of evolutionary allometry of flight energetics can help refine our understanding of links between flight form and function and how they may emerge from static allometry within species. Recent examples of the evolution of wing morphology in moth families and associated flight performance (Aiello et al. 2021) would likely have consequences on the evolution of metabolic properties (Bartholomew and Casey 1978).

The consistency of trait values obtained in species can help evaluate the strength of functional associations being studied, particularly for physiological measurements like MR. Flight MR measurements include errors due to variable flight states in a respirometry chamber, flight quality criteria, and measurement accuracy. Two data sets gathered on orchid bee species show the robustness of the macroevolutionary relationships obtained (Fig. 1D, E). With relatively modest sample sizes around 5−10 individuals, we found consistent scaling patterns and species differences, even for fine levels of residual variation (Fig. 1F). Using the variance component in a mixed-effects model accounting for body mass, the calculated intraclass correlation coefficient can be used to determine the repeatability of traits, and values are 0.89 and 0.60 for WBF and flight MR, respectively, showing that means obtained are highly reproducible, indicating that such species traits are robust. Overall, species morphological trait values force the flight function traits to be highly repeatable and representative of the species.

## Intraspecific variation and flight energetics

Macroevolutionary patterns result from microevolutionary mechanisms. Studying intraspecific scaling or static allometry can help understand the impact of body size on flight function and assess if conditions for adaptive phenotypic evolution are present. In species with determinate growth, body size and the phenotypes of interest have reduced variation, so the fit of regression and estimating parameters such as a scaling exponent can be more prone to error. To assess the relationship between body mass, wing size, and flight physiology, we have used the eusocial bumblebee *Bombus impatiens*, with access to many individuals and close to a five-fold range in worker body mass.

Wing area is proportional in *B. impatiens* workers. It has an exponent value of 0.668 (±0.016) (Fig. 1G), similar to values previously reported from other studies on this species (Buchwald and Dudley 2010; Skandalis and Darveau 2012). The wing size of large queens is also predicted from the worker’s regression, which supports the developmental program of isometric wing size to body size in this species (Billardon and Darveau 2019). The wing area’s isometric scaling allows testing the WBF prediction, which scales nearly exactly to the −1/6 power with an exponent value of −0.164 (±0.010) (Fig. 1H). Queens also fall directly within the predicted interval for most individuals (Fig. 3B). It is also important to note that WBF can still vary substantially for a given mass. Residual variation is correlated with wing area residual variation (Fig. 1I). Flight WBF within species scales with body mass due to the changes in wing size, which is isometric in this species. Slight deviations in wing size for a given body mass also impact WBF in a manner consistent with interspecific studies. Eusocial bumblebees also include male drones with slightly different body proportions. For a given mass, drones have slightly larger wings and lower WBF, as expected (Darveau et al. 2014). Similar sex dimorphism with lower wing loading for males is observed in several species of honey bees (Coelho 1991; Radloff et al. 2003), which may be associated with differences in flight performance. Differences in flight-related morphologies impact flight energetics, and variation in wing allometry between species and sexes is under a suite of evolutionary developmental constraints, environmental effects, and diverse selective pressures (Frankino et al. 2005; Shingleton et al. 2009; Dellicour et al. 2017; Shingleton and Frankino 2018; Houle et al. 2019; Le Roy et al. 2019; Rohner 2020; Rohner and Berger 2023), with apparent energetics consequences that should be explored further.

Intraspecific studies on other species can help test the predicted impact of body and wing size on WBF. The body mass range of leafcutter bees was extended to about 10-fold using different feeding regimes (Grula et al. 2021). Wing size scaling was hypoallometric with exponent values of *b* = 0.408 for wing area and *b* = 0.205 for wing length. Hypoallometric scaling of wing proportion is expected to reduce the dependence of WBF on body mass, which they observed with an absence of correlation. Developmental plasticity experiments can help shed light on the relationship between flight form and function. However, additional treatment effects during development can further impact flight physiology and performance, but it remains to be studied. Another example that highlights the importance of considering the proportions of the flight apparatus is the work conducted on carpenter bees (Roberts et al. 2004). Females of this species varying three-fold in body mass had hypoallometric wing area, thorax mass, and hyperallometric abdomen mass. None of the expected outcomes based on proportionally similar individuals hold in such cases; hence, the importance of the complete assessment of the central form-to-function relationship associated with insect flight. In the case of *B. impatiens*, thorax size is nearly isometric (Skandalis and Darveau 2012), which helps simplify the connections with Flight MR. Nonetheless, the study by Buchwald and Dudley (2010) indicates that flight muscle mass is hyperallometric in this species, so closer attention should be given to body and muscle proportions in such analysis.

Despite a fair amount of unexplained variance in flight MR among individuals, the static allometry follows the same association with WBF observed in evolutionary allometry. When expressed on a mass-specific basis, the static allometry of flight MR parallels WBF with an exponent *b* = −0.165 (±0.025) (Fig. 3B). The addition of queens on the same plot makes them fall on the worker’s regression line, further reinforcing the body and wing size to WBF and flight MR connection (Billardon and Darveau 2019). Drones that deviate from the females’ pattern with lower frequency for a given size also have lower flight MR (Darveau et al. 2014). Furthermore, the size-independent correlation between WBF and flight MR is found within species (Darveau et al. 2014; Billardon and Darveau 2019). This again reinforces that the associations between size, wing proportions, and flight energetics are quite conserved within species with caste-specific morphological phenotypes.

At the intraspecific level, the repeatability of individual traits has many uses in assessing the potential for adaptive phenotypic evolution, such as an indication of the upper limit of heritability of traits (Dohm 2002; Wilson 2018). The consistency of individual flight energetics has been assessed directly in the bumblebee *B. impatiens* workers (Darveau et al. 2014) and queens (Billardon and Darveau 2019). In other insect groups, active MR was also reported to be repeatable, such as in a species of butterfly (Niitepold and Hanski 2013), a system for which a significant heritability was also quantified (Mattila and Hanski 2014). This supports the idea that the family resemblance observed in *B. impatiens* (Billardon and Darveau 2019) may indicate that the phenotypic variance of flight-related traits is partly attributed to additive genetic variance. Hence, adaptive evolution may lead to the observed species differences in the trait cluster, including body size, wing size, WBF, and flight MR.

## Flight muscle metabolic phenotype changes with flight MR

Orchid bee species vary several-fold in flight MR, which allows us to determine some metabolic traits that may evolve with flight performance. Energy production during insect flight is strictly aerobic. In groups such as bees, it is thought to be fueled mainly through carbohydrates, although additional fuel such as the amino acid proline appears necessary (Suarez et al. 2005b; Teulier et al. 2016; Stec et al. 2021). The capacity for flux of biochemical pathways can be characterized by measuring the activity of enzymes, which is one means of regulating pathway flux (Suarez et al. 2005a). Looking at the activity of enzymes central to energy production pathways showed that specific enzymes covary with species flight MR, specifically some located at the entry point of the glycolytic pathway with hexokinase (HK), showing a tight correlation with orchid bee species flight MR (Fig. 3A). The parallel scaling of HK suggests a central role of this reaction in regulating overall pathway flux, which would explain the correlated evolution of this specific step and flight MR. Additional enzymes, including the connected steps catalyzed by trehalase and glycogen phosphorylase, also covary with flight MR but not to the same extent (Darveau et al. 2005a). In the model species *Drosophila melanogaster*, genetic manipulations to modulate the expression level of glycolytic enzymes show the strong impact of HK on flight capacity as measured by WBF (Eanes et al. 2006). The importance of this glycolytic step has helped connect the genetic basis of flight performance in *Drosophila* (Eanes 2011) and is central to the regulation of muscle glucose catabolism (Wasserman et al. 2011). Despite the complex regulation of energy metabolism, interindividual variation in flight MR is also most clearly associated with the activity of HK in *B. impatiens* workers (Fig. 3B), which is also correlated when looking at size-independent variation and differs between castes as predicted from the flight MR differences (Skandalis and Darveau 2012; Darveau et al. 2014; Billardon and Darveau 2019). This reinforces the hypothesis that microevolutionary processes can act on metabolic phenotypes and give rise to macroevolutionary diversity.

Insect flight muscle energy metabolism is diverse among groups with varying diets and the types of flight performed, and such complex phenotypes involve more than just a few regulatory steps. The extent to which flight MR relates to muscle oxidative capacity is still unclear in species such as bees. We seldom find correlations between flight MR and the activity of mitochondrial enzymes in orchid bees (Darveau et al. 2005a; Suarez et al. 2005b), and within species, it is not emerging as a strong association (Darveau et al. 2014). It has been suggested that in hymenopterans, the flight power requirement is related to the respiratory capacity of flight muscle fibers, but it is based on inferred and not measured flight power on three species (Hedges et al. 2019). Comparison of insect flight muscle oxidative capacity in varying species, such as bees and fruit flies (Menail et al. 2022), which differ substantially in flight MR (Niven and Scharlemann 2005), shows the challenges given the diverse arrangement of oxidative phosphorylation in flying insects. In butterflies, the association between flight MR and measures of oxidative capacity was shown within (Niitepõld et al. 2022) and across populations and species (Rauhamäki et al. 2014). Such important associations should be further scrutinized using empirically measured metabolic power and muscle mitochondrial oxidative properties across flying insects. The oxidative capacity is also in part associated with the phospholipids composition of cell membranes (Hulbert and Else 1999; Hulbert et al. 2002), which was shown to also covary with flight MR in the lineage of orchid bees (Rodríguez et al. 2015). Although a broad comparison shows how insects have pushed the muscle metabolic machinery to an apparent limit, including the central mitochondrial function (Suarez 2000; Bretscher and O'Connor 2020; Hickey et al. 2022), the extent to which flight MR necessarily evolves with mitochondrial function remains unclear.

**Fig. 4.**
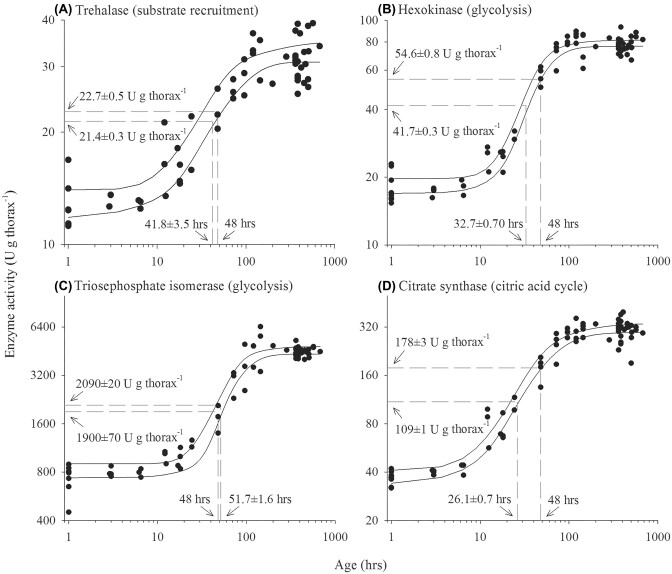
Maturation of the bumblebee *Bombus impatiens* flight muscle metabolic enzyme activity following adult emergence. The adult metabolic phenotype is set after about 4 days of maturation. The dashed lines represent the time to reach 50% of the adult activity and the activity after 48 h post-emergence. Solid lines represent the 95% confidence band of the regressions. Figure from Skandalis et al. (2011) with permission.

**Fig. 5.**
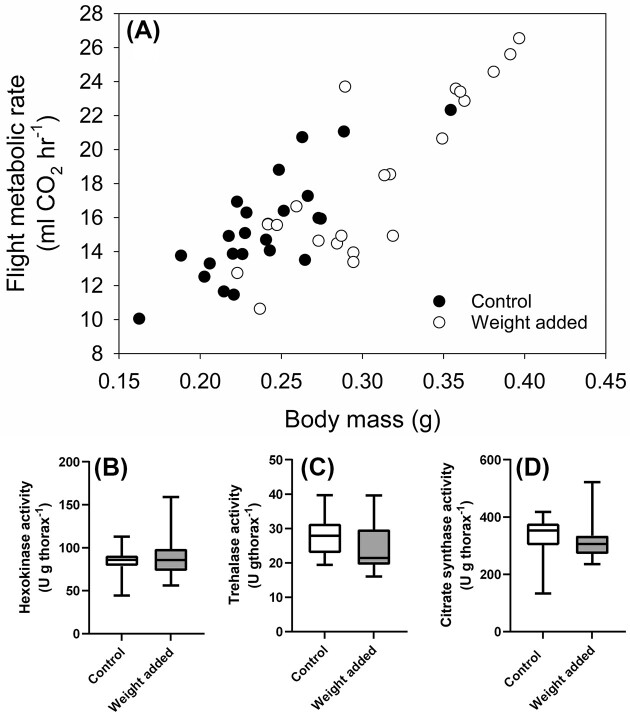
(A) Flight metabolic rate of *Bombus impatiens* workers that matured normally (●) and with a 40 mg weight affixed to their thorax (○) during the first day post-emergence. Flight metabolic rate was measured during trials conducted on days 5, 6, and 7 post-emergence (mean values shown). Using the individual’s native body mass, flight metabolic rate increased with body mass but did not change with added weight or consecutive flight trials (weight added: *F*_1,130_ = 2.159, *P* = 0.144; flight trial: *F*_2,130_ = 0.270, *P* = 0.763; native body mass: *F*_1,130_ = 131.5, *P* < 0.0001). Individuals were sampled on day 7 post-emergence, and the activity of the enzymes (B) hexokinase, (C) trehalase, and (D) citrate synthase was measured in the thorax. No increase in enzyme activity was found due to maturation with added weight, but a slightly lower trehalase activity was found in bees maturing with added weight (*P* = 0.041). Z. Corradini-Carriere and C.-A. Darveau, unpublished data.

The observed connections between body and wing size, flight MR, and flight muscle metabolic properties could be an outcome of the inherent plasticity of such tissue. Variations in individual size and wing proportion, which can be genetically determined and induced by environmental effects during development, can set energy demand and flight MR, which could be matched by muscle phenotype that can be labile and tuned to ATP demand. Flight muscle tissue appears very labile in *B. impatiens* that undergo large changes in flight muscle metabolic phenotype upon emergence. Adult workers have only about 25% of their flying adult enzyme activity at eclosion and reach their full potential after about four days postemergence (Fig. 4) (Skandalis et al. 2011). The potential for changes in flight muscle metabolism during the early maturational phase could represent an ideal window where flight form and function are tuned to each other. Flight muscle metabolic properties of adults can also change notably, as observed in honeybees during task transitions (Schippers et al. 2010) and age in other species (Wone et al. 2018; Fu et al. 2022). Flying insects also undergo changes in flight efforts and morphology during their lifetime, such as wing wear that can impact wing kinematics and flight performance (Hedenstrom et al. 2001; Haas and Cartar 2008; Combes et al. 2010). In *B. impatiens*, no difference in the activity of the muscle enzymes HK or trehalase could be detected with individual flight experience, flight restriction experiments, or experimentally induced wing wear (Skandalis and Darveau 2012), suggesting that metabolic phenotypes of adults are rigid in this species. To further test if flight muscle properties can change with additional load to lift during flight, we affixed added weight to workers’ thorax, corresponding to approximately 20% of the mean body mass, within their first day of emergence and before they are flight competent during the maturation period of the flight muscle. Despite the additional weight, flight MR did not increase and remained as predicted from the individual’s native body mass (Fig. 5A). Moreover, no detectable increase in the activity of HK, trehalase, and the mitochondrial enzyme citrate synthase occurred (Fig. 5B–D), indicating that no metabolic compensation took place with additional flight load. Flight muscle energy metabolism appears to be insensitive to variations in energy demand within an individual’s lifetime and may be set by the developmental program in such species. More work is needed to confirm how broadly this may apply and what features make insect muscle trainable to variable flight efforts.

## Perspective and future directions

**Fig. 6.**
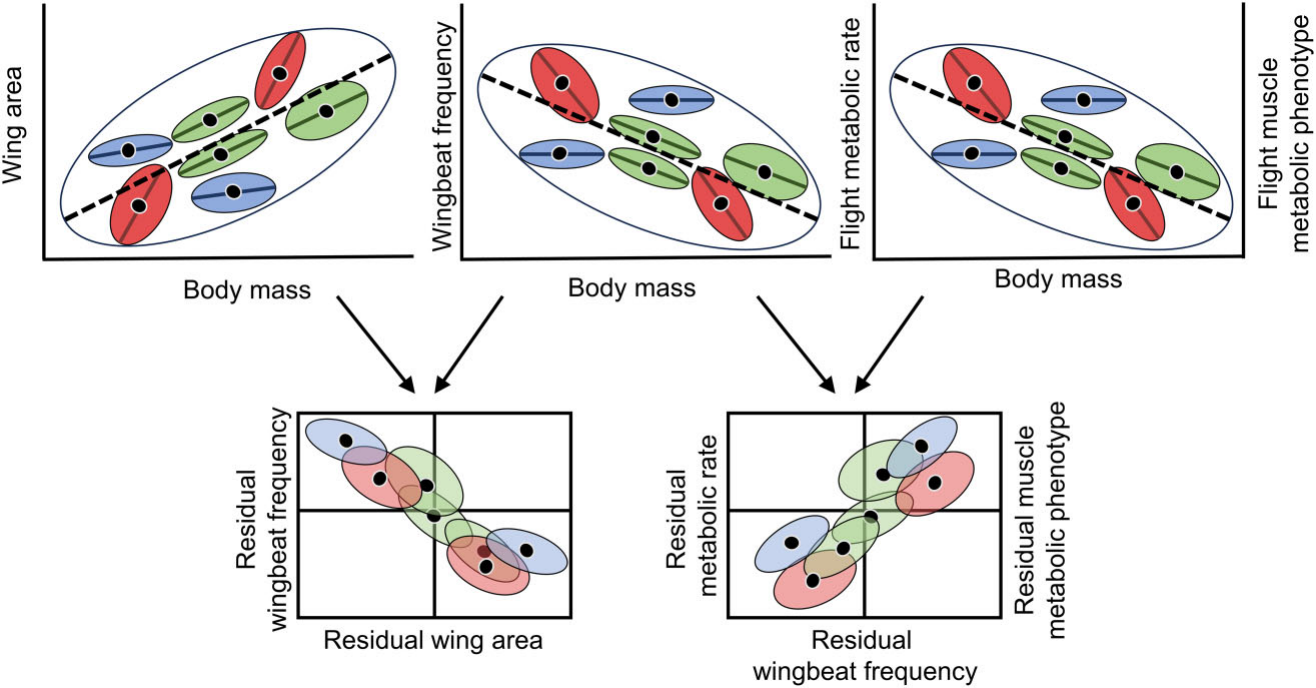
Representation of the relationship between static and evolutionary allometry of wing area, wingbeat frequency, flight metabolic rate, and flight muscle metabolic phenotype. Evolutionary allometry, represented by the large ellipses and dashed line, emerges from static allometries represented with species means (black dots). Smaller ellipses with solid lines represent intraspecific variation and static allometry. Wing area static allometry can be isometric or hyperallometric, leading to wingbeat frequency, flight metabolic rate, and flight muscle metabolic phenotype static allometry. Wing area static allometry can also be hypoallometric, leading to only slight or absent static allometry of flight energetics parameters. Size-independent variation in wing proportions, within and across species, explains size-independent deviations in wingbeat frequency, which explains size-independent variation in flight metabolic rate and flight muscle metabolic phenotype. Overall, variable static allometry in wing size ultimately impacts or not flight muscle metabolic properties and evolutionary allometry patterns will emerge depending on the species range and static allometry present.

The diversity of insect wing size and shape is vast, and understanding the functional determinants of the morphospace occupied by certain species or groups is still a central challenge (Le Roy et al. 2019; Salcedo et al. 2019; Rohner 2020; Aiello et al. 2021). We know that wing size allometry is evolvable, but strong selection forces species-specific wing allometry (Frankino et al. 2005; Bolstad et al. 2015; Houle et al. 2019). Pinpointing the ultimate reasons for wing size, shape, and proportions remains challenging due to the complexity of factors affecting flight performance, developmental constraints, and the contribution of various selective pressures, including sexual selection. The current work does not address the ultimate “why” of insect wing size and shape but instead explains the series of functional outcomes of the diverse allometric association between body and wing size. Using the presented functional associations, we can assess the proximate mechanisms leading to the diversity of insect flight energetics based on static and evolutionary allometry of wing size. As summarized in Fig. 6, the information obtained on static allometry within species indicates that wing allometry trajectory (hypo, iso, or hyperallometry) can influence the variation in flight WBF. This, in turn, explains flight MR scaling and the associated flight muscle metabolic phenotype. Variation along the body mass axis can be explained, but deviations independent of body size follow the same functional links. The microevolutionary process acting on the observed phenotypic variation can ultimately lead to speciation, where varying allometry may exist. However, looking at species mean, the same relationships can be described with varying outcomes depending on the observed morphospace occupied and wing size scaling. Hence, the concept of morphospace use in evolutionary developmental biology can be extended to comparative physiology, where a connected physiospace emerges and forms these macroevolutionary flight energetics patterns. The whole-animal function, such as WBF and the classical MR scaling, can be explained from morphological scaling, but also cellular metabolic properties that permit flight function and associated variation. Insect flight energetics provides a biological system to further our understanding of the evolution of size, form, and function. Future efforts will incorporate the influence of environmental conditions during development on modifying body and wing proportions and sexual dimorphism and, ultimately, use experimental evolution to assess the physiological impact of morphological evolution.

## Data Availability

The data underlying this article will be shared on reasonable request to the corresponding author.

## Notes

From the symposium “Evolution, Physiology, and Biomechanics of Insect Flight” presented at the annual meeting of the Society for Integrative and Comparative Biology, January 2–6, 2024 in Seattle.
